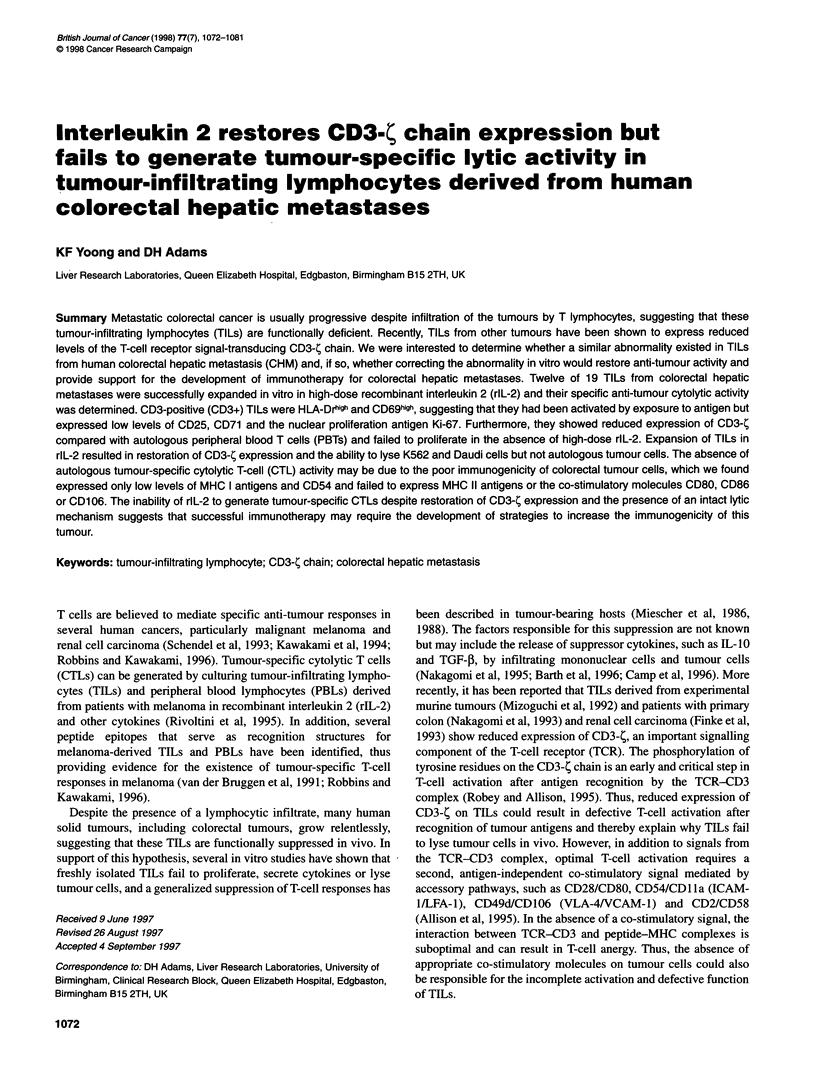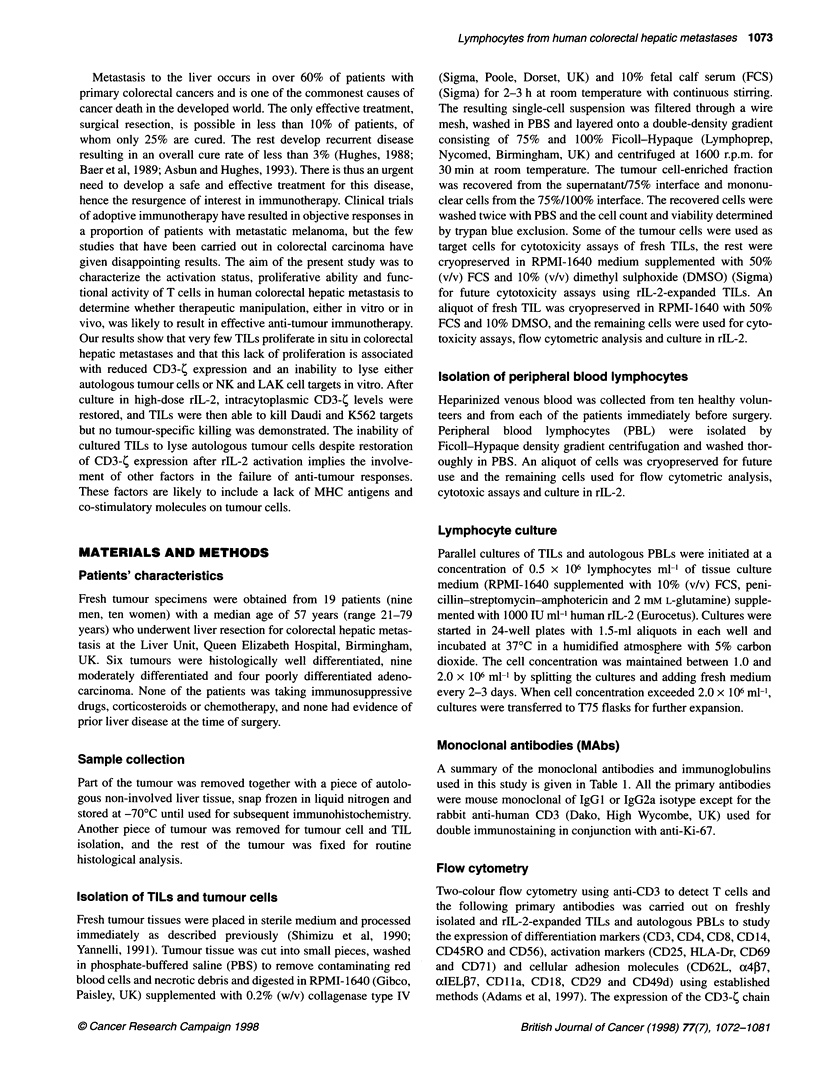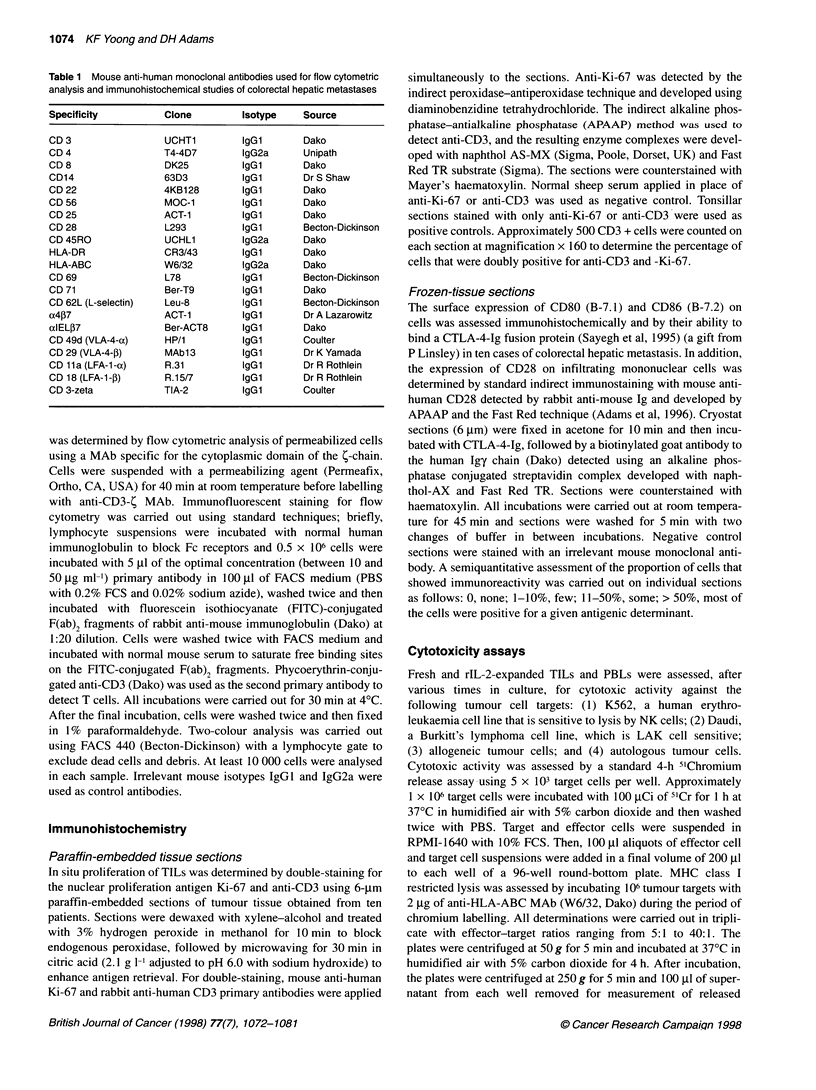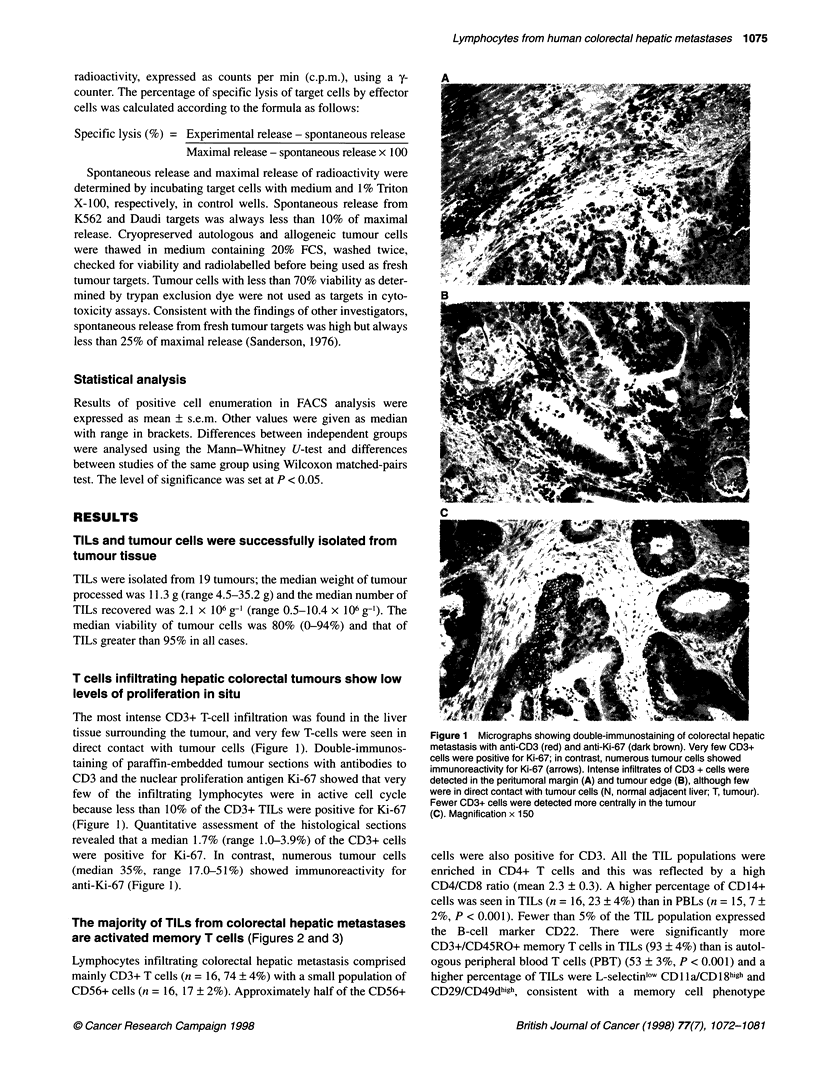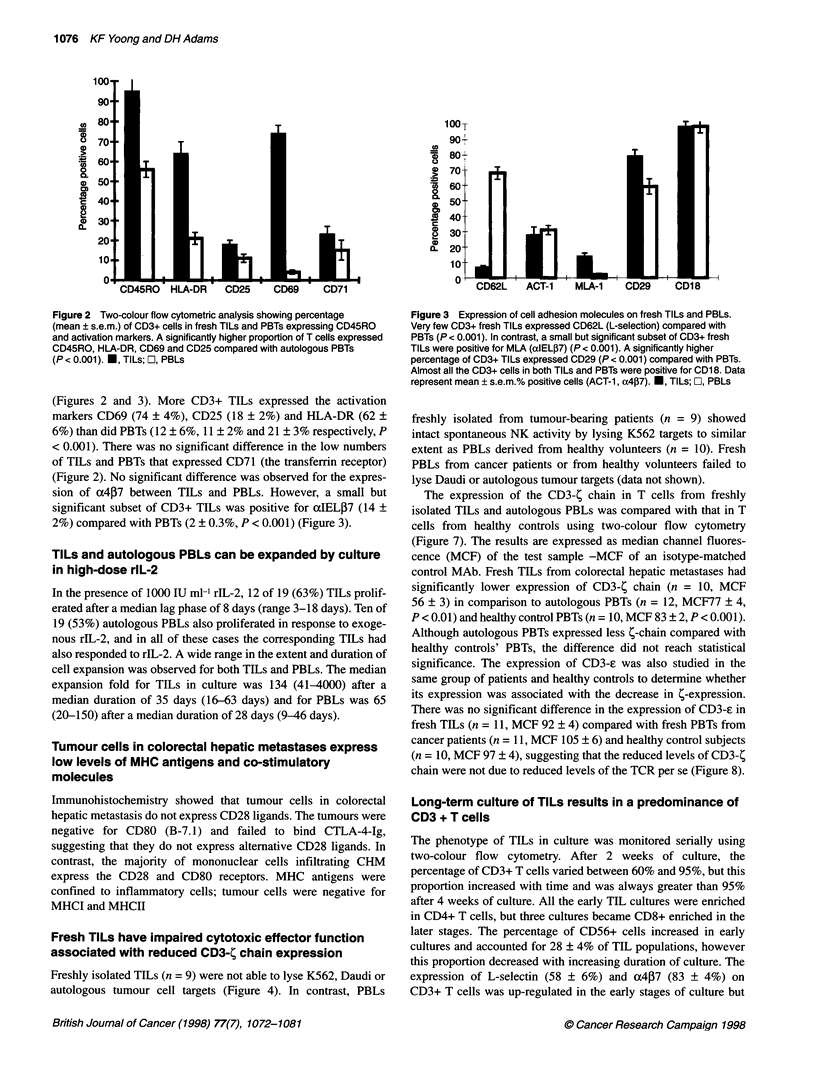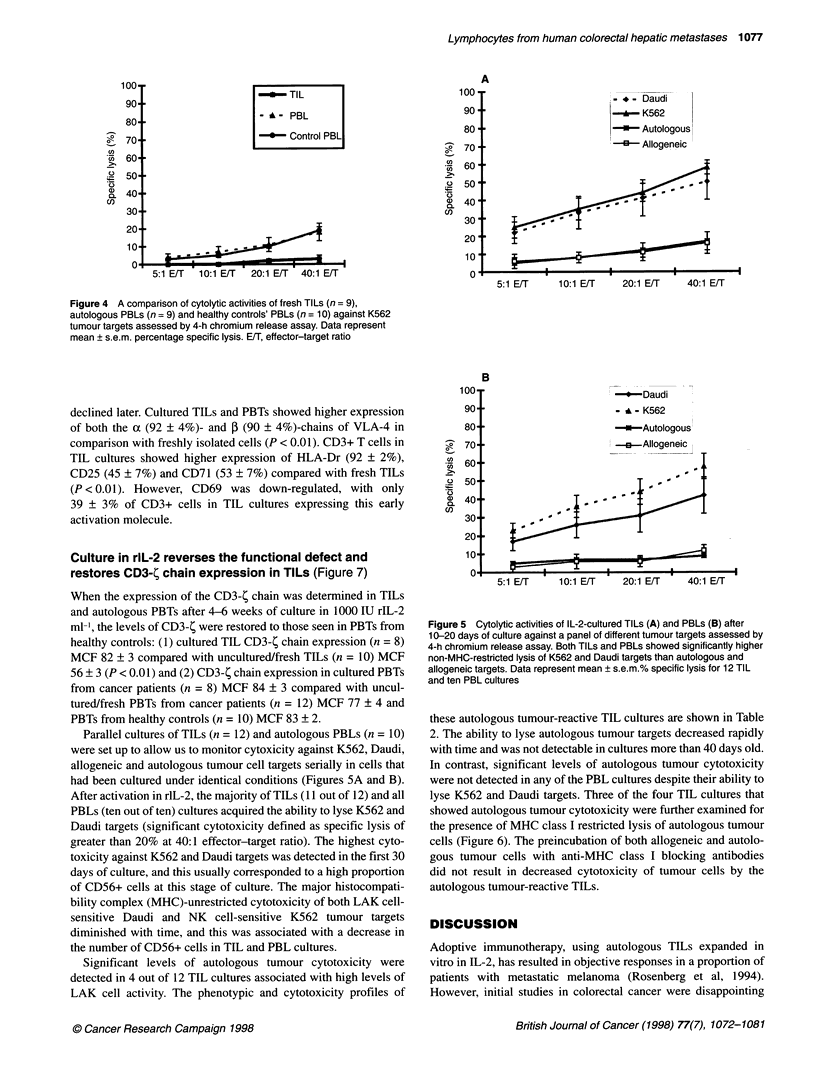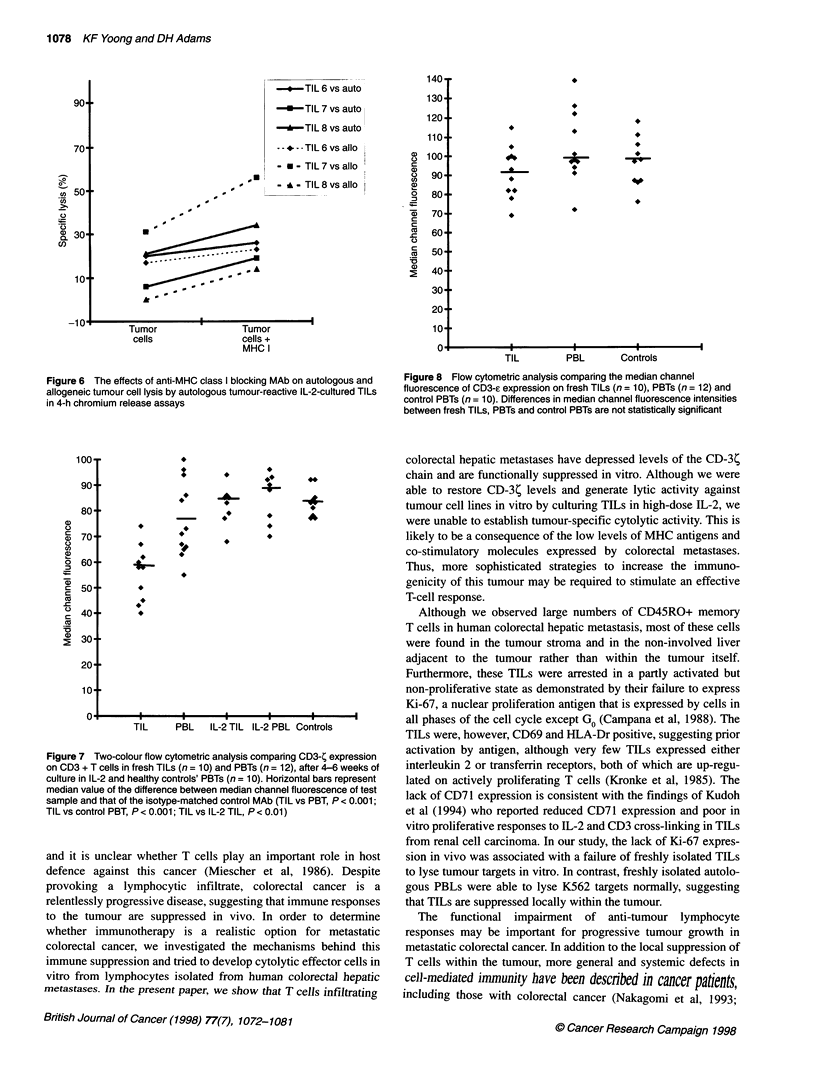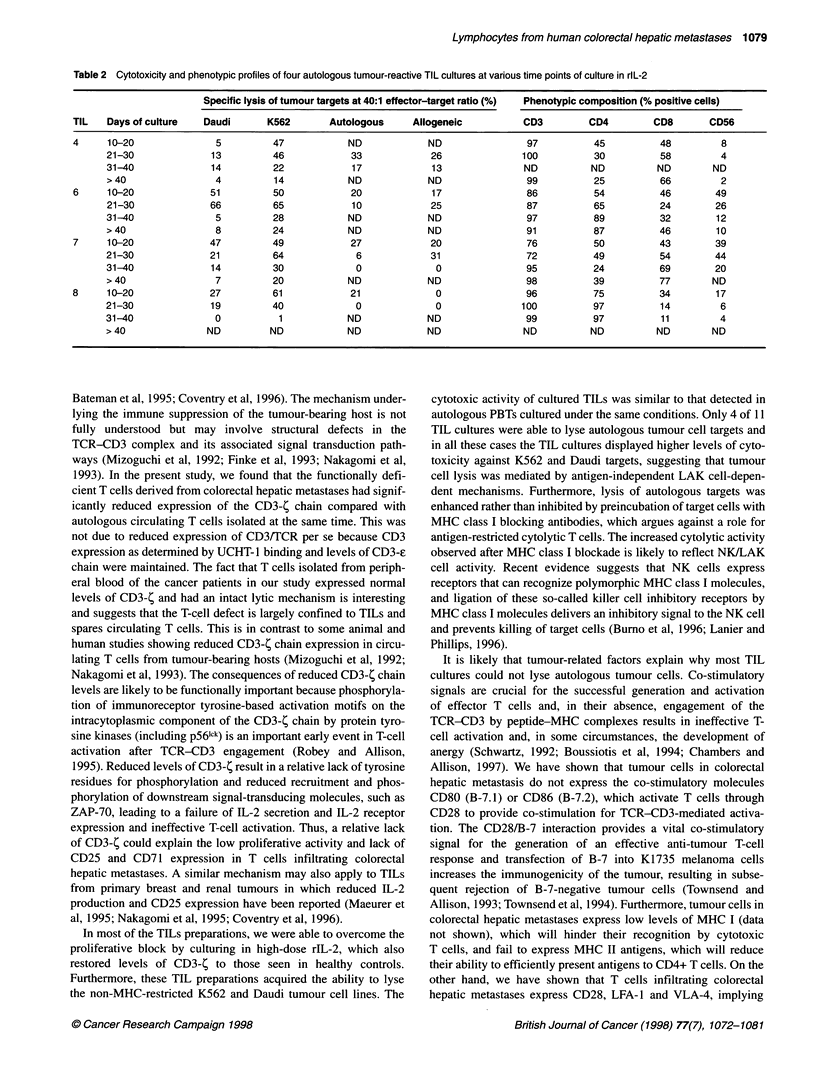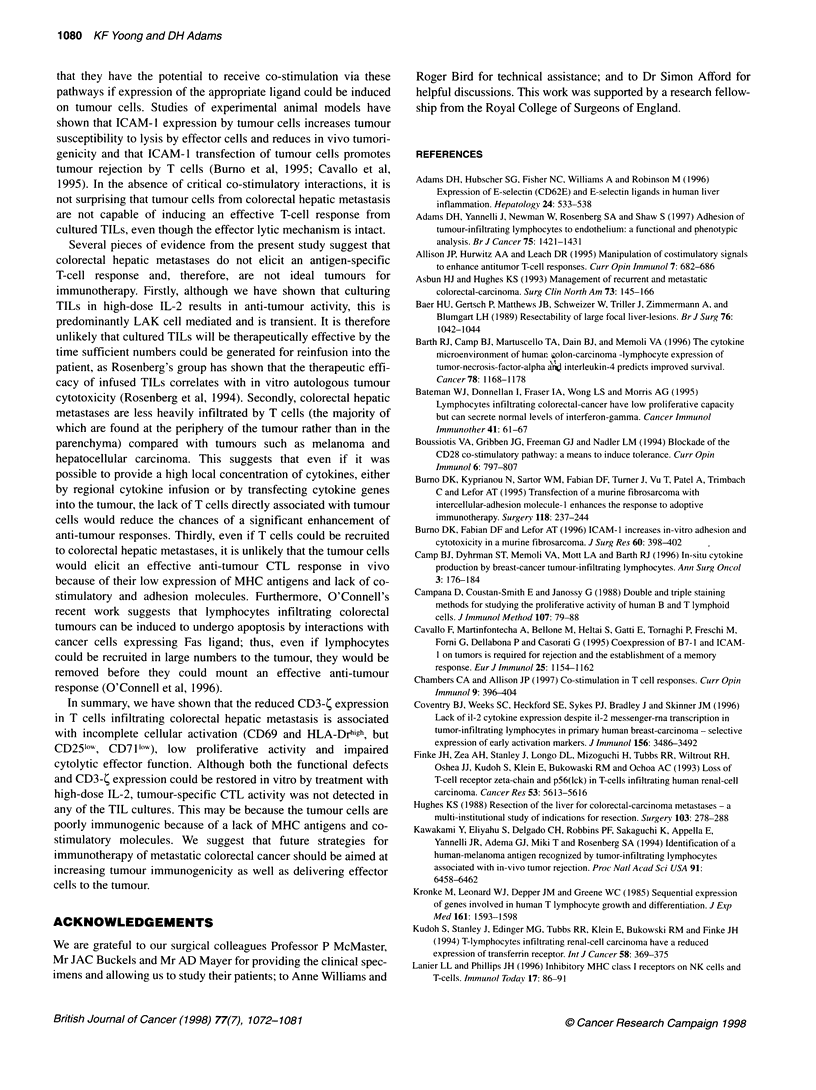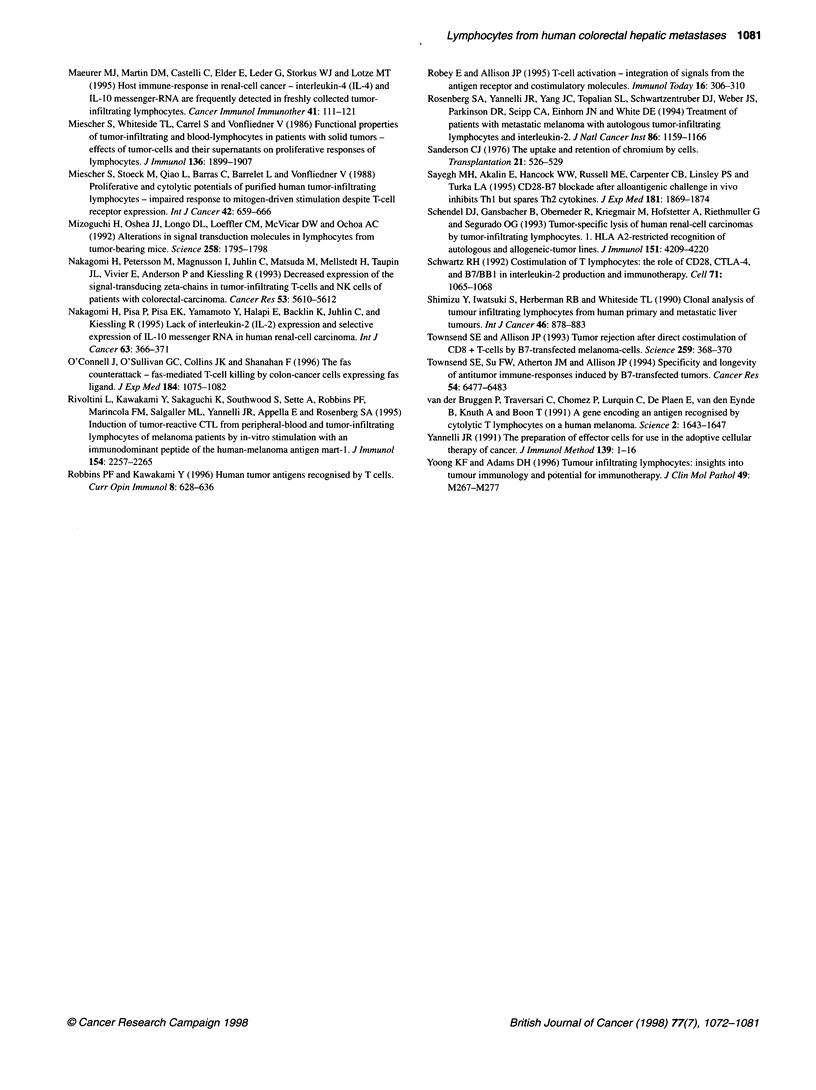# Interleukin 2 restores CD3-zeta chain expression but fails to generate tumour-specific lytic activity in tumour-infiltrating lymphocytes derived from human colorectal hepatic metastases.

**DOI:** 10.1038/bjc.1998.179

**Published:** 1998-04

**Authors:** K. F. Yoong, D. H. Adams

**Affiliations:** Liver Research Laboratories, Queen Elizabeth Hospital, Edgbaston, Birmingham, UK.

## Abstract

**Images:**


British Journal of Cancer (1998) 77(7), 1072–1081
© 1998 Cancer Research Campaign

### Interleukin 2 restores CD3-ζ chain expression but fails to generate tumour-specific lytic activity in tumour-infiltrating lymphocytes derived from human colorectal hepatic metastases

**KF Yoong and DH Adams**

Liver Research Laboratories, Queen Elizabeth Hospital, Edgbaston, Birmingham B15 2TH, UK

**Summary** Metastatic colorectal cancer is usually progressive despite infiltration of the tumours by T lymphocytes, suggesting that these tumour-infiltrating lymphocytes (TILs) are functionally deficient. Recently, TILs from other tumours have been shown to express reduced levels of the T-cell receptor signal-transducing CD3-ζ chain. We were interested to determine whether a similar abnormality existed in TILs from human colorectal hepatic metastasis (CHM) and, if so, whether correcting the abnormality in vitro would restore anti-tumour activity and provide support for the development of immunotherapy for colorectal hepatic metastases. Twelve of 19 TILs from colorectal hepatic metastases were successfully expanded in vitro in high-dose recombinant interleukin 2 (rIL-2) and their specific anti-tumour cytolytic activity was determined. CD3-positive (CD3+) TILs were HLA-Dr$^{high}$ and CD69$^{high}$, suggesting that they had been activated by exposure to antigen but expressed low levels of CD25, CD71 and the nuclear proliferation antigen Ki-67. Furthermore, they showed reduced expression of CD3-ζ compared with autologous peripheral blood T cells (PBTs) and failed to proliferate in the absence of high-dose rIL-2. Expansion of TILs in rIL-2 resulted in restoration of CD3-ζ expression and the ability to lyse K562 and Daudi cells but not autologous tumour cells. The absence of autologous tumour-specific cytolytic T-cell (CTL) activity may be due to the poor immunogenicity of colorectal tumour cells, which we found expressed only low levels of MHC I antigens and CD54 and failed to express MHC II antigens or the co-stimulatory molecules CD80, CD86 or CD106. The inability of rIL-2 to generate tumour-specific CTLs despite restoration of CD3-ζ expression and the presence of an intact lytic mechanism suggests that successful immunotherapy may require the development of strategies to increase the immunogenicity of this tumour.

**Keywords:** tumour-infiltrating lymphocyte; CD3-ζ chain; colorectal hepatic metastasis

T cells are believed to mediate specific anti-tumour responses in several human cancers, particularly malignant melanoma and renal cell carcinoma (Schendel et al, 1993; Kawakami et al, 1994; Robbins and Kawakami, 1996). Tumour-specific cytolytic T cells (CTLs) can be generated by culturing tumour-infiltrating lymphocytes (TILs) and peripheral blood lymphocytes (PBLs) derived from patients with melanoma in recombinant interleukin 2 (rIL-2) and other cytokines (Rivoltini et al, 1995). In addition, several peptide epitopes that serve as recognition structures for melanoma-derived TILs and PBLs have been identified, thus providing evidence for the existence of tumour-specific T-cell responses in melanoma (van der Bruggen et al, 1991; Robbins and Kawakami, 1996).

Despite the presence of a lymphocytic infiltrate, many human solid tumours, including colorectal tumours, grow relentlessly, suggesting that these TILs are functionally suppressed in vivo. In support of this hypothesis, several in vitro studies have shown that freshly isolated TILs fail to proliferate, secrete cytokines or lyse tumour cells, and a generalized suppression of T-cell responses has been described in tumour-bearing hosts (Miescher et al, 1986, 1988). The factors responsible for this suppression are not known but may include the release of suppressor cytokines, such as IL-10 and TGF-β, by infiltrating mononuclear cells and tumour cells (Nakagomi et al, 1995; Barth et al, 1996; Camp et al, 1996). More recently, it has been reported that TILs derived from experimental murine tumours (Mizoguchi et al, 1992) and patients with primary colon (Nakagomi et al, 1993) and renal cell carcinoma (Finke et al, 1993) show reduced expression of CD3-ζ, an important signalling component of the T-cell receptor (TCR). The phosphorylation of tyrosine residues on the CD3-ζ chain is an early and critical step in T-cell activation after antigen recognition by the TCR–CD3 complex (Robey and Allison, 1995). Thus, reduced expression of CD3-ζ on TILs could result in defective T-cell activation after recognition of tumour antigens and thereby explain why TILs fail to lyse tumour cells in vivo. However, in addition to signals from the TCR–CD3 complex, optimal T-cell activation requires a second, antigen-independent co-stimulatory signal mediated by accessory pathways, such as CD28/CD80, CD54/CD11a (ICAM-1/LFA-1), CD49d/CD106 (VLA-4/VCAM-1) and CD2/CD58 (Allison et al, 1995). In the absence of a co-stimulatory signal, the interaction between TCR–CD3 and peptide–MHC complexes is suboptimal and can result in T-cell anergy. Thus, the absence of appropriate co-stimulatory molecules on tumour cells could also be responsible for the incomplete activation and defective function of TILs.

*Received 9 June 1997*
*Revised 26 August 1997*
*Accepted 4 September 1997*

*Correspondence to:* DH Adams, Liver Research Laboratories, University of Birmingham, Clinical Research Block, Queen Elizabeth Hospital, Edgbaston, Birmingham B15 2TH, UK

Metastasis to the liver occurs in over 60% of patients with primary colorectal cancers and is one of the commonest causes of cancer death in the developed world. The only effective treatment, surgical resection, is possible in less than 10% of patients, of whom only 25% are cured. The rest develop recurrent disease resulting in an overall cure rate of less than 3% (Hughes, 1988; Baer et al, 1989; Asbun and Hughes, 1993). There is thus an urgent need to develop a safe and effective treatment for this disease, hence the resurgence of interest in immunotherapy. Clinical trials of adoptive immunotherapy have resulted in objective responses in a proportion of patients with metastatic melanoma, but the few studies that have been carried out in colorectal carcinoma have given disappointing results. The aim of the present study was to characterize the activation status, proliferative ability and functional activity of T cells in human colorectal hepatic metastasis to determine whether therapeutic manipulation, either in vitro or in vivo, was likely to result in effective anti-tumour immunotherapy. Our results show that very few TILs proliferate in situ in colorectal hepatic metastases and that this lack of proliferation is associated with reduced CD3-ζ expression and an inability to lyse either autologous tumour cells or NK and LAK cell targets in vitro. After culture in high-dose rIL-2, intracytoplasmic CD3-ζ levels were restored, and TILs were then able to kill Daudi and K562 targets but no tumour-specific killing was demonstrated. The inability of cultured TILs to lyse autologous tumour cells despite restoration of CD3-ζ expression after rIL-2 activation implies the involvement of other factors in the failure of anti-tumour responses. These factors are likely to include a lack of MHC antigens and co-stimulatory molecules on tumour cells.

### MATERIALS AND METHODS

### Patients' characteristics

Fresh tumour specimens were obtained from 19 patients (nine men, ten women) with a median age of 57 years (range 21–79 years) who underwent liver resection for colorectal hepatic metastasis at the Liver Unit, Queen Elizabeth Hospital, Birmingham, UK. Six tumours were histologically well differentiated, nine moderately differentiated and four poorly differentiated adenocarcinoma. None of the patients was taking immunosuppressive drugs, corticosteroids or chemotherapy, and none had evidence of prior liver disease at the time of surgery.

### Sample collection

Part of the tumour was removed together with a piece of autologous non-involved liver tissue, snap frozen in liquid nitrogen and stored at –70°C until used for subsequent immunohistochemistry. Another piece of tumour was removed for tumour cell and TIL isolation, and the rest of the tumour was fixed for routine histological analysis.

### Isolation of TILs and tumour cells

Fresh tumour tissues were placed in sterile medium and processed immediately as described previously (Shimizu et al, 1990; Yannelli, 1991). Tumour tissue was cut into small pieces, washed in phosphate-buffered saline (PBS) to remove contaminating red blood cells and necrotic debris and digested in RPMI-1640 (Gibco, Paisley, UK) supplemented with 0.2% (w/v) collagenase type IV (Sigma, Poole, Dorset, UK) and 10% fetal calf serum (FCS) (Sigma) for 2–3 h at room temperature with continuous stirring. The resulting single-cell suspension was filtered through a wire mesh, washed in PBS and layered onto a double-density gradient consisting of 75% and 100% Ficoll–Hypaque (Lymphoprep, Nycomed, Birmingham, UK) and centrifuged at 1600 r.p.m. for 30 min at room temperature. The tumour cell-enriched fraction was recovered from the supernatant/75% interface and mononuclear cells from the 75%/100% interface. The recovered cells were washed twice with PBS and the cell count and viability determined by trypan blue exclusion. Some of the tumour cells were used as target cells for cytotoxicity assays of fresh TILs, the rest were cryopreserved in RPMI-1640 medium supplemented with 50% (v/v) FCS and 10% (v/v) dimethyl sulphoxide (DMSO) (Sigma) for future cytotoxicity assays using rIL-2-expanded TILs. An aliquot of fresh TIL was cryopreserved in RPMI-1640 with 50% FCS and 10% DMSO, and the remaining cells were used for cytotoxicity assays, flow cytometric analysis and culture in rIL-2.

### Isolation of peripheral blood lymphocytes

Heparinized venous blood was collected from ten healthy volunteers and from each of the patients immediately before surgery. Peripheral blood lymphocytes (PBL) were isolated by Ficoll–Hypaque density gradient centrifugation and washed thoroughly in PBS. An aliquot of cells was cryopreserved for future use and the remaining cells used for flow cytometric analysis, cytotoxic assays and culture in rIL-2.

### Lymphocyte culture

Parallel cultures of TILs and autologous PBLs were initiated at a concentration of $0.5 \times 10^6$ lymphocytes $ml^{-1}$ of tissue culture medium (RPMI-1640 supplemented with 10% (v/v) FCS, penicillin–streptomycin–amphotericin and 2 mM L-glutamine) supplemented with 1000 IU $ml^{-1}$ human rIL-2 (Eurocetus). Cultures were started in 24-well plates with 1.5-ml aliquots in each well and incubated at 37°C in a humidified atmosphere with 5% carbon dioxide. The cell concentration was maintained between 1.0 and $2.0 \times 10^6$ $ml^{-1}$ by splitting the cultures and adding fresh medium every 2–3 days. When cell concentration exceeded $2.0 \times 10^6$ $ml^{-1}$, cultures were transferred to T75 flasks for further expansion.

### Monoclonal antibodies (MAbs)

A summary of the monoclonal antibodies and immunoglobulins used in this study is given in Table 1. All the primary antibodies were mouse monoclonal of IgG1 or IgG2a isotype except for the rabbit anti-human CD3 (Dako, High Wycombe, UK) used for double immunostaining in conjunction with anti-Ki-67.

### Flow cytometry

Two-colour flow cytometry using anti-CD3 to detect T cells and the following primary antibodies was carried out on freshly isolated and rIL-2-expanded TILs and autologous PBLs to study the expression of differentiation markers (CD3, CD4, CD8, CD14, CD45RO and CD56), activation markers (CD25, HLA-Dr, CD69 and CD71) and cellular adhesion molecules (CD62L, α4β7, αIELβ7, CD11a, CD18, CD29 and CD49d) using established methods (Adams et al, 1997). The expression of the CD3-ζ chain

© Cancer Research Campaign 1998

**Table 1** Mouse anti-human monoclonal antibodies used for flow cytometric analysis and immunohistochemical studies of colorectal hepatic metastases

| Specificity | Clone | Isotype | Source |
|---|---|---|---|
| CD 3 | UCHT1 | IgG1 | Dako |
| CD 4 | T4-4D7 | IgG2a | Unipath |
| CD 8 | DK25 | IgG1 | Dako |
| CD14 | 63D3 | IgG1 | Dr S Shaw |
| CD 22 | 4KB128 | IgG1 | Dako |
| CD 56 | MOC-1 | IgG1 | Dako |
| CD 25 | ACT-1 | IgG1 | Dako |
| CD 28 | L293 | IgG1 | Becton-Dickinson |
| CD 45RO | UCHL1 | IgG2a | Dako |
| HLA-DR | CR3/43 | IgG1 | Dako |
| HLA-ABC | W6/32 | IgG2a | Dako |
| CD 69 | L78 | IgG1 | Becton-Dickinson |
| CD 71 | Ber-T9 | IgG1 | Dako |
| CD 62L (L-selectin) | Leu-8 | IgG1 | Becton-Dickinson |
| α4β7 | ACT-1 | IgG1 | Dr A Lazarowitz |
| αIELβ7 | Ber-ACT8 | IgG1 | Dako |
| CD 49d (VLA-4-α) | HP/1 | IgG1 | Coulter |
| CD 29 (VLA-4-β) | MAb13 | IgG1 | Dr K Yamada |
| CD 11a (LFA-1-α) | R.31 | IgG1 | Dr R Rothlein |
| CD 18 (LFA-1-β) | R.15/7 | IgG1 | Dr R Rothlein |
| CD 3-zeta | TIA-2 | IgG1 | Coulter |

was determined by flow cytometric analysis of permeabilized cells using a MAb specific for the cytoplasmic domain of the ζ-chain. Cells were suspended with a permeabilizing agent (Permeafix, Ortho, CA, USA) for 40 min at room temperature before labelling with anti-CD3-ζ MAb. Immunofluorescent staining for flow cytometry was carried out using standard techniques; briefly, lymphocyte suspensions were incubated with normal human immunoglobulin to block Fc receptors and $0.5 \times 10^6$ cells were incubated with 5 μl of the optimal concentration (between 10 and 50 μg $ml^{-1}$) primary antibody in 100 μl of FACS medium (PBS with 0.2% FCS and 0.02% sodium azide), washed twice and then incubated with fluorescein isothiocyanate (FITC)-conjugated $F(ab)_2$ fragments of rabbit anti-mouse immunoglobulin (Dako) at 1:20 dilution. Cells were washed twice with FACS medium and incubated with normal mouse serum to saturate free binding sites on the FITC-conjugated $F(ab)_2$ fragments. Phycoerythrin-conjugated anti-CD3 (Dako) was used as the second primary antibody to detect T cells. All incubations were carried out for 30 min at 4°C. After the final incubation, cells were washed twice and then fixed in 1% paraformaldehyde. Two-colour analysis was carried out using FACS 440 (Becton-Dickinson) with a lymphocyte gate to exclude dead cells and debris. At least 10 000 cells were analysed in each sample. Irrelevant mouse isotypes IgG1 and IgG2a were used as control antibodies.

### Immunohistochemistry

### *Paraffin-embedded tissue sections*

In situ proliferation of TILs was determined by double-staining for the nuclear proliferation antigen Ki-67 and anti-CD3 using 6-μm paraffin-embedded sections of tumour tissue obtained from ten patients. Sections were dewaxed with xylene–alcohol and treated with 3% hydrogen peroxide in methanol for 10 min to block endogenous peroxidase, followed by microwaving for 30 min in citric acid (2.1 g $l^{-1}$ adjusted to pH 6.0 with sodium hydroxide) to enhance antigen retrieval. For double-staining, mouse anti-human Ki-67 and rabbit anti-human CD3 primary antibodies were applied simultaneously to the sections. Anti-Ki-67 was detected by the indirect peroxidase–antiperoxidase technique and developed using diaminobenzidine tetrahydrochloride. The indirect alkaline phosphatase–antialkaline phosphatase (APAAP) method was used to detect anti-CD3, and the resulting enzyme complexes were developed with naphthol AS-MX (Sigma, Poole, Dorset, UK) and Fast Red TR substrate (Sigma). The sections were counterstained with Mayer's haematoxylin. Normal sheep serum applied in place of anti-Ki-67 or anti-CD3 was used as negative control. Tonsillar sections stained with only anti-Ki-67 or anti-CD3 were used as positive controls. Approximately 500 CD3 + cells were counted on each section at magnification × 160 to determine the percentage of cells that were doubly positive for anti-CD3 and -Ki-67.

### *Frozen-tissue sections*

The surface expression of CD80 (B-7.1) and CD86 (B-7.2) on cells was assessed immunohistochemically and by their ability to bind a CTLA-4-Ig fusion protein (Sayegh et al, 1995) (a gift from P Linsley) in ten cases of colorectal hepatic metastasis. In addition, the expression of CD28 on infiltrating mononuclear cells was determined by standard indirect immunostaining with mouse anti-human CD28 detected by rabbit anti-mouse Ig and developed by APAAP and the Fast Red technique (Adams et al, 1996). Cryostat sections (6 μm) were fixed in acetone for 10 min and then incubated with CTLA-4-Ig, followed by a biotinylated goat antibody to the human Igγ chain (Dako) detected using an alkaline phosphatase conjugated streptavidin complex developed with naphthol-AX and Fast Red TR. Sections were counterstained with haematoxylin. All incubations were carried out at room temperature for 45 min and sections were washed for 5 min with two changes of buffer in between incubations. Negative control sections were stained with an irrelevant mouse monoclonal antibody. A semiquantitative assessment of the proportion of cells that showed immunoreactivity was carried out on individual sections as follows: 0, none; 1–10%, few; 11–50%, some; > 50%, most of the cells were positive for a given antigenic determinant.

### Cytotoxicity assays

Fresh and rIL-2-expanded TILs and PBLs were assessed, after various times in culture, for cytotoxic activity against the following tumour cell targets: (1) K562, a human erythroleukaemia cell line that is sensitive to lysis by NK cells; (2) Daudi, a Burkitt's lymphoma cell line, which is LAK cell sensitive; (3) allogeneic tumour cells; and (4) autologous tumour cells. Cytotoxic activity was assessed by a standard 4-h $^{51}$Chromium release assay using $5 \times 10^3$ target cells per well. Approximately $1 \times 10^6$ target cells were incubated with 100 μCi of $^{51}Cr$ for 1 h at 37°C in humidified air with 5% carbon dioxide and then washed twice with PBS. Target and effector cells were suspended in RPMI-1640 with 10% FCS. Then, 100 μl aliquots of effector cell and target cell suspensions were added in a final volume of 200 μl to each well of a 96-well round-bottom plate. MHC class I restricted lysis was assessed by incubating $10^6$ tumour targets with 2 μg of anti-HLA-ABC MAb (W6/32, Dako) during the period of chromium labelling. All determinations were carried out in triplicate with effector–target ratios ranging from 5:1 to 40:1. The plates were centrifuged at 50 ***g*** for 5 min and incubated at 37°C in humidified air with 5% carbon dioxide for 4 h. After incubation, the plates were centrifuged at 250 ***g*** for 5 min and 100 μl of supernatant from each well removed for measurement of released

© Cancer Research Campaign 1998

radioactivity, expressed as counts per min (c.p.m.), using a γ-counter. The percentage of specific lysis of target cells by effector cells was calculated according to the formula as follows:

$$\text{Specific lysis (\%)} = \frac{\text{Experimental release} - \text{spontaneous release}}{\text{Maximal release} - \text{spontaneous release} \times 100}$$

Spontaneous release and maximal release of radioactivity were determined by incubating target cells with medium and 1% Triton X-100, respectively, in control wells. Spontaneous release from K562 and Daudi targets was always less than 10% of maximal release. Cryopreserved autologous and allogeneic tumour cells were thawed in medium containing 20% FCS, washed twice, checked for viability and radiolabelled before being used as fresh tumour targets. Tumour cells with less than 70% viability as determined by trypan exclusion dye were not used as targets in cytotoxicity assays. Consistent with the findings of other investigators, spontaneous release from fresh tumour targets was high but always less than 25% of maximal release (Sanderson, 1976).

### Statistical analysis

Results of positive cell enumeration in FACS analysis were expressed as mean ± s.e.m. Other values were given as median with range in brackets. Differences between independent groups were analysed using the Mann–Whitney *U*-test and differences between studies of the same group using Wilcoxon matched-pairs test. The level of significance was set at $P < 0.05$.

### RESULTS

### TILs and tumour cells were successfully isolated from tumour tissue

TILs were isolated from 19 tumours; the median weight of tumour processed was 11.3 g (range 4.5–35.2 g) and the median number of TILs recovered was $2.1 \times 10^6$ g$^{-1}$ (range $0.5–10.4 \times 10^6$ g$^{-1}$). The median viability of tumour cells was 80% (0–94%) and that of TILs greater than 95% in all cases.

### T cells infiltrating hepatic colorectal tumours show low levels of proliferation in situ

The most intense CD3+ T-cell infiltration was found in the liver tissue surrounding the tumour, and very few T-cells were seen in direct contact with tumour cells (Figure 1). Double-immunostaining of paraffin-embedded tumour sections with antibodies to CD3 and the nuclear proliferation antigen Ki-67 showed that very few of the infiltrating lymphocytes were in active cell cycle because less than 10% of the CD3+ TILs were positive for Ki-67 (Figure 1). Quantitative assessment of the histological sections revealed that a median 1.7% (range 1.0–3.9%) of the CD3+ cells were positive for Ki-67. In contrast, numerous tumour cells (median 35%, range 17.0–51%) showed immunoreactivity for anti-Ki-67 (Figure 1).

### The majority of TILs from colorectal hepatic metastases are activated memory T cells (Figures 2 and 3)

Lymphocytes infiltrating colorectal hepatic metastasis comprised mainly CD3+ T cells ($n = 16$, 74 ± 4%) with a small population of CD56+ cells ($n = 16$, 17 ± 2%). Approximately half of the CD56+ cells were also positive for CD3. All the TIL populations were enriched in CD4+ T cells and this was reflected by a high CD4/CD8 ratio (mean 2.3 ± 0.3). A higher percentage of CD14+ cells was seen in TILs ($n = 16$, 23 ± 4%) than in PBLs ($n = 15$, 7 ± 2%, $P < 0.001$). Fewer than 5% of the TIL population expressed the B-cell marker CD22. There were significantly more CD3+/CD45RO+ memory T cells in TILs (93 ± 4%) than is autologous peripheral blood T cells (PBT) (53 ± 3%, $P < 0.001$) and a higher percentage of TILs were L-selectin$^{low}$ CD11a/CD18$^{high}$ and CD29/CD49d$^{high}$, consistent with a memory cell phenotype

**Figure 1** Micrographs showing double-immunostaining of colorectal hepatic metastasis with anti-CD3 (red) and anti-Ki-67 (dark brown). Very few CD3+ cells were positive for Ki-67; in contrast, numerous tumour cells showed immunoreactivity for Ki-67 (arrows). Intense infiltrates of CD3 + cells were detected in the peritumoral margin (**A**) and tumour edge (**B**), although few were in direct contact with tumour cells (N, normal adjacent liver; T, tumour). Fewer CD3+ cells were detected more centrally in the tumour (**C**). Magnification × 150

**Figure 2** Two-colour flow cytometric analysis showing percentage (mean ± s.e.m.) of CD3+ cells in fresh TILs and PBTs expressing CD45RO and activation markers. A significantly higher proportion of T cells expressed CD45RO, HLA-DR, CD69 and CD25 compared with autologous PBTs ($P < 0.001$). ■, TILs; □, PBLs

**Figure 3** Expression of cell adhesion molecules on fresh TILs and PBLs. Very few CD3+ fresh TILs expressed CD62L (L-selection) compared with PBTs ($P < 0.001$). In contrast, a small but significant subset of CD3+ fresh TILs were positive for MLA (αIELβ7) ($P < 0.001$). A significantly higher percentage of CD3+ TILs expressed CD29 ($P < 0.001$) compared with PBTs. Almost all the CD3+ cells in both TILs and PBTs were positive for CD18. Data represent mean ± s.e.m.% positive cells (ACT-1, α4β7). ■, TILs; □, PBLs

(Figures 2 and 3). More CD3+ TILs expressed the activation markers CD69 (74 ± 4%), CD25 (18 ± 2%) and HLA-DR (62 ± 6%) than did PBTs (12 ± 6%, 11 ± 2% and 21 ± 3% respectively, $P < 0.001$). There was no significant difference in the low numbers of TILs and PBTs that expressed CD71 (the transferrin receptor) (Figure 2). No significant difference was observed for the expression of α4β7 between TILs and PBLs. However, a small but significant subset of CD3+ TILs was positive for αIELβ7 (14 ± 2%) compared with PBTs (2 ± 0.3%, $P < 0.001$) (Figure 3).

### TILs and autologous PBLs can be expanded by culture in high-dose rIL-2

In the presence of 1000 IU $ml^{-1}$ rIL-2, 12 of 19 (63%) TILs proliferated after a median lag phase of 8 days (range 3–18 days). Ten of 19 (53%) autologous PBLs also proliferated in response to exogenous rIL-2, and in all of these cases the corresponding TILs had also responded to rIL-2. A wide range in the extent and duration of cell expansion was observed for both TILs and PBLs. The median expansion fold for TILs in culture was 134 (41–4000) after a median duration of 35 days (16–63 days) and for PBLs was 65 (20–150) after a median duration of 28 days (9–46 days).

### Tumour cells in colorectal hepatic metastases express low levels of MHC antigens and co-stimulatory molecules

Immunohistochemistry showed that tumour cells in colorectal hepatic metastasis do not express CD28 ligands. The tumours were negative for CD80 (B-7.1) and failed to bind CTLA-4-Ig, suggesting that they do not express alternative CD28 ligands. In contrast, the majority of mononuclear cells infiltrating CHM express the CD28 and CD80 receptors. MHC antigens were confined to inflammatory cells; tumour cells were negative for MHCI and MHCII

### Fresh TILs have impaired cytotoxic effector function associated with reduced CD3-ζ chain expression

Freshly isolated TILs ($n = 9$) were not able to lyse K562, Daudi or autologous tumour cell targets (Figure 4). In contrast, PBLs freshly isolated from tumour-bearing patients ($n = 9$) showed intact spontaneous NK activity by lysing K562 targets to similar extent as PBLs derived from healthy volunteers ($n = 10$). Fresh PBLs from cancer patients or from healthy volunteers failed to lyse Daudi or autologous tumour targets (data not shown).

The expression of the CD3-ζ chain in T cells from freshly isolated TILs and autologous PBLs was compared with that in T cells from healthy controls using two-colour flow cytometry (Figure 7). The results are expressed as median channel fluorescence (MCF) of the test sample –MCF of an isotype-matched control MAb. Fresh TILs from colorectal hepatic metastases had significantly lower expression of CD3-ζ chain ($n = 10$, MCF 56 ± 3) in comparison to autologous PBTs ($n = 12$, MCF77 ± 4, $P < 0.01$) and healthy control PBTs ($n = 10$, MCF 83 ± 2, $P < 0.001$). Although autologous PBTs expressed less ζ-chain compared with healthy controls' PBTs, the difference did not reach statistical significance. The expression of CD3-ε was also studied in the same group of patients and healthy controls to determine whether its expression was associated with the decrease in ζ-expression. There was no significant difference in the expression of CD3-ε in fresh TILs ($n = 11$, MCF 92 ± 4) compared with fresh PBTs from cancer patients ($n = 11$, MCF 105 ± 6) and healthy control subjects ($n = 10$, MCF 97 ± 4), suggesting that the reduced levels of CD3-ζ chain were not due to reduced levels of the TCR per se (Figure 8).

### Long-term culture of TILs results in a predominance of CD3 + T cells

The phenotype of TILs in culture was monitored serially using two-colour flow cytometry. After 2 weeks of culture, the percentage of CD3+ T cells varied between 60% and 95%, but this proportion increased with time and was always greater than 95% after 4 weeks of culture. All the early TIL cultures were enriched in CD4+ T cells, but three cultures became CD8+ enriched in the later stages. The percentage of CD56+ cells increased in early cultures and accounted for 28 ± 4% of TIL populations, however this proportion decreased with increasing duration of culture. The expression of L-selectin (58 ± 6%) and α4β7 (83 ± 4%) on CD3+ T cells was up-regulated in the early stages of culture but

© *Cancer Research Campaign 1998*

**Figure 4** A comparison of cytolytic activities of fresh TILs ($n = 9$), autologous PBLs ($n = 9$) and healthy controls' PBLs ($n = 10$) against K562 tumour targets assessed by 4-h chromium release assay. Data represent mean ± s.e.m. percentage specific lysis. E/T, effector–target ratio

**Figure 5** Cytolytic activities of IL-2-cultured TILs (**A**) and PBLs (**B**) after 10–20 days of culture against a panel of different tumour targets assessed by 4-h chromium release assay. Both TILs and PBLs showed significantly higher non-MHC-restricted lysis of K562 and Daudi targets than autologous and allogeneic targets. Data represent mean ± s.e.m.% specific lysis for 12 TIL and ten PBL cultures declined later. Cultured TILs and PBTs showed higher expression of both the α (92 ± 4%)- and β (90 ± 4%)-chains of VLA-4 in comparison with freshly isolated cells ($P < 0.01$). CD3+ T cells in TIL cultures showed higher expression of HLA-Dr (92 ± 2%), CD25 (45 ± 7%) and CD71 (53 ± 7%) compared with fresh TILs ($P < 0.01$). However, CD69 was down-regulated, with only 39 ± 3% of CD3+ cells in TIL cultures expressing this early activation molecule.

### Culture in rIL-2 reverses the functional defect and restores CD3-ζ chain expression in TILs (Figure 7)

When the expression of the CD3-ζ chain was determined in TILs and autologous PBTs after 4–6 weeks of culture in 1000 IU rIL-2 $ml^{-1}$, the levels of CD3-ζ were restored to those seen in PBTs from healthy controls: (1) cultured TIL CD3-ζ chain expression ($n = 8$) MCF 82 ± 3 compared with uncultured/fresh TILs ($n = 10$) MCF 56 ± 3 ($P < 0.01$) and (2) CD3-ζ chain expression in cultured PBTs from cancer patients ($n = 8$) MCF 84 ± 3 compared with uncultured/fresh PBTs from cancer patients ($n = 12$) MCF 77 ± 4 and PBTs from healthy controls ($n = 10$) MCF 83 ± 2.

Parallel cultures of TILs ($n = 12$) and autologous PBLs ($n = 10$) were set up to allow us to monitor cytoxicity against K562, Daudi, allogeneic and autologous tumour cell targets serially in cells that had been cultured under identical conditions (Figures 5A and B). After activation in rIL-2, the majority of TILs (11 out of 12) and all PBLs (ten out of ten) cultures acquired the ability to lyse K562 and Daudi targets (significant cytotoxicity defined as specific lysis of greater than 20% at 40:1 effector–target ratio). The highest cytotoxicity against K562 and Daudi targets was detected in the first 30 days of culture, and this usually corresponded to a high proportion of CD56+ cells at this stage of culture. The major histocompatibility complex (MHC)-unrestricted cytotoxicity of both LAK cell-sensitive Daudi and NK cell-sensitive K562 tumour targets diminished with time, and this was associated with a decrease in the number of CD56+ cells in TIL and PBL cultures.

Significant levels of autologous tumour cytotoxicity were detected in 4 out of 12 TIL cultures associated with high levels of LAK cell activity. The phenotypic and cytotoxicity profiles of these autologous tumour-reactive TIL cultures are shown in Table 2. The ability to lyse autologous tumour targets decreased rapidly with time and was not detectable in cultures more than 40 days old. In contrast, significant levels of autologous tumour cytotoxicity were not detected in any of the PBL cultures despite their ability to lyse K562 and Daudi targets. Three of the four TIL cultures that showed autologous tumour cytotoxicity were further examined for the presence of MHC class I restricted lysis of autologous tumour cells (Figure 6). The preincubation of both allogeneic and autologous tumour cells with anti-MHC class I blocking antibodies did not result in decreased cytotoxicity of tumour cells by the autologous tumour-reactive TILs.

### DISCUSSION

Adoptive immunotherapy, using autologous TILs expanded in vitro in IL-2, has resulted in objective responses in a proportion of patients with metastatic melanoma (Rosenberg et al, 1994). However, initial studies in colorectal cancer were disappointing

**Figure 6** The effects of anti-MHC class I blocking MAb on autologous and allogeneic tumour cell lysis by autologous tumour-reactive IL-2-cultured TILs in 4-h chromium release assays

**Figure 8** Flow cytometric analysis comparing the median channel fluorescence of CD3-ε expression on fresh TILs ($n$ = 10), PBTs ($n$ = 12) and control PBTs ($n$ = 10). Differences in median channel fluorescence intensities between fresh TILs, PBTs and control PBTs are not statistically significant

**Figure 7** Two-colour flow cytometric analysis comparing CD3-ζ expression on CD3 + T cells in fresh TILs ($n$ = 10) and PBTs ($n$ = 12), after 4–6 weeks of culture in IL-2 and healthy controls' PBTs ($n$ = 10). Horizontal bars represent median value of the difference between median channel fluorescence of test sample and that of the isotype-matched control MAb (TIL vs PBT, $P < 0.001$; TIL vs control PBT, $P < 0.001$; TIL vs IL-2 TIL, $P < 0.01$)

and it is unclear whether T cells play an important role in host defence against this cancer (Miescher et al, 1986). Despite provoking a lymphocytic infiltrate, colorectal cancer is a relentlessly progressive disease, suggesting that immune responses to the tumour are suppressed in vivo. In order to determine whether immunotherapy is a realistic option for metastatic colorectal cancer, we investigated the mechanisms behind this immune suppression and tried to develop cytolytic effector cells in vitro from lymphocytes isolated from human colorectal hepatic metastases. In the present paper, we show that T cells infiltrating colorectal hepatic metastases have depressed levels of the CD-3ζ chain and are functionally suppressed in vitro. Although we were able to restore CD-3ζ levels and generate lytic activity against tumour cell lines in vitro by culturing TILs in high-dose IL-2, we were unable to establish tumour-specific cytolytic activity. This is likely to be a consequence of the low levels of MHC antigens and co-stimulatory molecules expressed by colorectal metastases. Thus, more sophisticated strategies to increase the immunogenicity of this tumour may be required to stimulate an effective T-cell response.

Although we observed large numbers of CD45RO+ memory T cells in human colorectal hepatic metastasis, most of these cells were found in the tumour stroma and in the non-involved liver adjacent to the tumour rather than within the tumour itself. Furthermore, these TILs were arrested in a partly activated but non-proliferative state as demonstrated by their failure to express Ki-67, a nuclear proliferation antigen that is expressed by cells in all phases of the cell cycle except $G_0$ (Campana et al, 1988). The TILs were, however, CD69 and HLA-Dr positive, suggesting prior activation by antigen, although very few TILs expressed either interleukin 2 or transferrin receptors, both of which are up-regulated on actively proliferating T cells (Kronke et al, 1985). The lack of CD71 expression is consistent with the findings of Kudoh et al (1994) who reported reduced CD71 expression and poor in vitro proliferative responses to IL-2 and CD3 cross-linking in TILs from renal cell carcinoma. In our study, the lack of Ki-67 expression in vivo was associated with a failure of freshly isolated TILs to lyse tumour targets in vitro. In contrast, freshly isolated autologous PBLs were able to lyse K562 targets normally, suggesting that TILs are suppressed locally within the tumour.

The functional impairment of anti-tumour lymphocyte responses may be important for progressive tumour growth in metastatic colorectal cancer. In addition to the local suppression of T cells within the tumour, more general and systemic defects in cell-mediated immunity have been described in cancer patients, including those with colorectal cancer (Nakagomi et al, 1993;

© Cancer Research Campaign 1998

**Table 2** Cytotoxicity and phenotypic profiles of four autologous tumour-reactive TIL cultures at various time points of culture in rIL-2

| | | Specific lysis of tumour targets at 40:1 effector–target ratio (%) | | | | Phenotypic composition (% positive cells) | | | |
|---|---|---|---|---|---|---|---|---|---|
| TIL | Days of culture | Daudi | K562 | Autologous | Allogeneic | CD3 | CD4 | CD8 | CD56 |
| 4 | 10–20 | 5 | 47 | ND | ND | 97 | 45 | 48 | 8 |
| | 21–30 | 13 | 46 | 33 | 26 | 100 | 30 | 58 | 4 |
| | 31–40 | 14 | 22 | 17 | 13 | ND | ND | ND | ND |
| | > 40 | 4 | 14 | ND | ND | 99 | 25 | 66 | 2 |
| 6 | 10–20 | 51 | 50 | 20 | 17 | 86 | 54 | 46 | 49 |
| | 21–30 | 66 | 65 | 10 | 25 | 87 | 65 | 24 | 26 |
| | 31–40 | 5 | 28 | ND | ND | 97 | 89 | 32 | 12 |
| | > 40 | 8 | 24 | ND | ND | 91 | 87 | 46 | 10 |
| 7 | 10–20 | 47 | 49 | 27 | 20 | 76 | 50 | 43 | 39 |
| | 21–30 | 21 | 64 | 6 | 31 | 72 | 49 | 54 | 44 |
| | 31–40 | 14 | 30 | 0 | 0 | 95 | 24 | 69 | 20 |
| | > 40 | 7 | 20 | ND | ND | 98 | 39 | 77 | ND |
| 8 | 10–20 | 27 | 61 | 21 | 0 | 96 | 75 | 34 | 17 |
| | 21–30 | 19 | 40 | 0 | 0 | 100 | 97 | 14 | 6 |
| | 31–40 | 0 | 1 | ND | ND | 99 | 97 | 11 | 4 |
| | > 40 | ND | ND | ND | ND | ND | ND | ND | ND |

Bateman et al, 1995; Coventry et al, 1996). The mechanism underlying the immune suppression of the tumour-bearing host is not fully understood but may involve structural defects in the TCR–CD3 complex and its associated signal transduction pathways (Mizoguchi et al, 1992; Finke et al, 1993; Nakagomi et al, 1993). In the present study, we found that the functionally deficient T cells derived from colorectal hepatic metastases had significantly reduced expression of the CD3-ζ chain compared with autologous circulating T cells isolated at the same time. This was not due to reduced expression of CD3/TCR per se because CD3 expression as determined by UCHT-1 binding and levels of CD3-ε chain were maintained. The fact that T cells isolated from peripheral blood of the cancer patients in our study expressed normal levels of CD3-ζ and had an intact lytic mechanism is interesting and suggests that the T-cell defect is largely confined to TILs and spares circulating T cells. This is in contrast to some animal and human studies showing reduced CD3-ζ chain expression in circulating T cells from tumour-bearing hosts (Mizoguchi et al, 1992; Nakagomi et al, 1993). The consequences of reduced CD3-ζ chain levels are likely to be functionally important because phosphorylation of immunoreceptor tyrosine-based activation motifs on the intracytoplasmic component of the CD3-ζ chain by protein tyrosine kinases (including $p56^{lck}$) is an important early event in T-cell activation after TCR–CD3 engagement (Robey and Allison, 1995). Reduced levels of CD3-ζ result in a relative lack of tyrosine residues for phosphorylation and reduced recruitment and phosphorylation of downstream signal-transducing molecules, such as ZAP-70, leading to a failure of IL-2 secretion and IL-2 receptor expression and ineffective T-cell activation. Thus, a relative lack of CD3-ζ could explain the low proliferative activity and lack of CD25 and CD71 expression in T cells infiltrating colorectal hepatic metastases. A similar mechanism may also apply to TILs from primary breast and renal tumours in which reduced IL-2 production and CD25 expression have been reported (Maeurer et al, 1995; Nakagomi et al, 1995; Coventry et al, 1996).

In most of the TILs preparations, we were able to overcome the proliferative block by culturing in high-dose rIL-2, which also restored levels of CD3-ζ to those seen in healthy controls. Furthermore, these TIL preparations acquired the ability to lyse the non-MHC-restricted K562 and Daudi tumour cell lines. The cytotoxic activity of cultured TILs was similar to that detected in autologous PBTs cultured under the same conditions. Only 4 of 11 TIL cultures were able to lyse autologous tumour cell targets and in all these cases the TIL cultures displayed higher levels of cytotoxicity against K562 and Daudi targets, suggesting that tumour cell lysis was mediated by antigen-independent LAK cell-dependent mechanisms. Furthermore, lysis of autologous targets was enhanced rather than inhibited by preincubation of target cells with MHC class I blocking antibodies, which argues against a role for antigen-restricted cytolytic T cells. The increased cytolytic activity observed after MHC class I blockade is likely to reflect NK/LAK cell activity. Recent evidence suggests that NK cells express receptors that can recognize polymorphic MHC class I molecules, and ligation of these so-called killer cell inhibitory receptors by MHC class I molecules delivers an inhibitory signal to the NK cell and prevents killing of target cells (Burno et al, 1996; Lanier and Phillips, 1996).

It is likely that tumour-related factors explain why most TIL cultures could not lyse autologous tumour cells. Co-stimulatory signals are crucial for the successful generation and activation of effector T cells and, in their absence, engagement of the TCR–CD3 by peptide–MHC complexes results in ineffective T-cell activation and, in some circumstances, the development of anergy (Schwartz, 1992; Boussiotis et al, 1994; Chambers and Allison, 1997). We have shown that tumour cells in colorectal hepatic metastasis do not express the co-stimulatory molecules CD80 (B-7.1) or CD86 (B-7.2), which activate T cells through CD28 to provide co-stimulation for TCR–CD3-mediated activation. The CD28/B-7 interaction provides a vital co-stimulatory signal for the generation of an effective anti-tumour T-cell response and transfection of B-7 into K1735 melanoma cells increases the immunogenicity of the tumour, resulting in subsequent rejection of B-7-negative tumour cells (Townsend and Allison, 1993; Townsend et al, 1994). Furthermore, tumour cells in colorectal hepatic metastases express low levels of MHC I (data not shown), which will hinder their recognition by cytotoxic T cells, and fail to express MHC II antigens, which will reduce their ability to efficiently present antigens to CD4+ T cells. On the other hand, we have shown that T cells infiltrating colorectal hepatic metastases express CD28, LFA-1 and VLA-4, implying that they have the potential to receive co-stimulation via these pathways if expression of the appropriate ligand could be induced on tumour cells. Studies of experimental animal models have shown that ICAM-1 expression by tumour cells increases tumour susceptibility to lysis by effector cells and reduces in vivo tumorigenicity and that ICAM-1 transfection of tumour cells promotes tumour rejection by T cells (Burno et al, 1995; Cavallo et al, 1995). In the absence of critical co-stimulatory interactions, it is not surprising that tumour cells from colorectal hepatic metastasis are not capable of inducing an effective T-cell response from cultured TILs, even though the effector lytic mechanism is intact.

Several pieces of evidence from the present study suggest that colorectal hepatic metastases do not elicit an antigen-specific T-cell response and, therefore, are not ideal tumours for immunotherapy. Firstly, although we have shown that culturing TILs in high-dose IL-2 results in anti-tumour activity, this is predominantly LAK cell mediated and is transient. It is therefore unlikely that cultured TILs will be therapeutically effective by the time sufficient numbers could be generated for reinfusion into the patient, as Rosenberg's group has shown that the therapeutic efficacy of infused TILs correlates with in vitro autologous tumour cytotoxicity (Rosenberg et al, 1994). Secondly, colorectal hepatic metastases are less heavily infiltrated by T cells (the majority of which are found at the periphery of the tumour rather than in the parenchyma) compared with tumours such as melanoma and hepatocellular carcinoma. This suggests that even if it was possible to provide a high local concentration of cytokines, either by regional cytokine infusion or by transfecting cytokine genes into the tumour, the lack of T cells directly associated with tumour cells would reduce the chances of a significant enhancement of anti-tumour responses. Thirdly, even if T cells could be recruited to colorectal hepatic metastases, it is unlikely that the tumour cells would elicit an effective anti-tumour CTL response in vivo because of their low expression of MHC antigens and lack of co-stimulatory and adhesion molecules. Furthermore, O'Connell's recent work suggests that lymphocytes infiltrating colorectal tumours can be induced to undergo apoptosis by interactions with cancer cells expressing Fas ligand; thus, even if lymphocytes could be recruited in large numbers to the tumour, they would be removed before they could mount an effective anti-tumour response (O'Connell et al, 1996).

In summary, we have shown that the reduced CD3-ζ expression in T cells infiltrating colorectal hepatic metastasis is associated with incomplete cellular activation (CD69 and HLA-$Dr^{high}$, but $CD25^{low}$, $CD71^{low}$), low proliferative activity and impaired cytolytic effector function. Although both the functional defects and CD3-ζ expression could be restored in vitro by treatment with high-dose IL-2, tumour-specific CTL activity was not detected in any of the TIL cultures. This may be because the tumour cells are poorly immunogenic because of a lack of MHC antigens and co-stimulatory molecules. We suggest that future strategies for immunotherapy of metastatic colorectal cancer should be aimed at increasing tumour immunogenicity as well as delivering effector cells to the tumour.

### ACKNOWLEDGEMENTS

We are grateful to our surgical colleagues Professor P McMaster, Mr JAC Buckels and Mr AD Mayer for providing the clinical specimens and allowing us to study their patients; to Anne Williams and Roger Bird for technical assistance; and to Dr Simon Afford for helpful discussions. This work was supported by a research fellowship from the Royal College of Surgeons of England.

### REFERENCES

Adams DH, Hubscher SG, Fisher NC, Williams A and Robinson M (1996) Expression of E-selectin (CD62E) and E-selectin ligands in human liver inflammation. *Hepatology* **24**: 533–538

Adams DH, Yannelli J, Newman W, Rosenberg SA and Shaw S (1997) Adhesion of tumour-infiltrating lymphocytes to endothelium: a functional and phenotypic analysis. *Br J Cancer* **75**: 1421–1431

Allison JP, Hurwitz AA and Leach DR (1995) Manipulation of costimulatory signals to enhance antitumor T-cell responses. *Curr Opin Immunol* **7**: 682–686

Asbun HJ and Hughes KS (1993) Management of recurrent and metastatic colorectal-carcinoma. *Surg Clin North Am* **73**: 145–166

Baer HU, Gertsch P, Matthews JB, Schweizer W, Triller J, Zimmermann A, and Blumgart LH (1989) Resectability of large focal liver-lesions. *Br J Surg* **76**: 1042–1044

Barth RJ, Camp BJ, Martuscello TA, Dain BJ, and Memoli VA (1996) The cytokine microenvironment of human colon-carcinoma -lymphocyte expression of tumor-necrosis-factor-alpha and interleukin-4 predicts improved survival. *Cancer* **78**: 1168–1178

Bateman WJ, Donnellan I, Fraser IA, Wong LS and Morris AG (1995) Lymphocytes infiltrating colorectal-cancer have low proliferative capacity but can secrete normal levels of interferon-gamma. *Cancer Immunol Immunother* **41**: 61–67

Boussiotis VA, Gribben JG, Freeman GJ and Nadler LM (1994) Blockade of the CD28 co-stimulatory pathway: a means to induce tolerance. *Curr Opin Immunol* **6**: 797–807

Burno DK, Kyprianou N, Sartor WM, Fabian DF, Turner J, Vu T, Patel A, Trimbach C and Lefor AT (1995) Transfection of a murine fibrosarcoma with intercellular-adhesion molecule-1 enhances the response to adoptive immunotherapy. *Surgery* **118**: 237–244

Burno DK, Fabian DF and Lefor AT (1996) ICAM-1 increases in-vitro adhesion and cytotoxicity in a murine fibrosarcoma. *J Surg Res* **60**: 398–402

Camp BJ, Dyhrman ST, Memoli VA, Mott LA and Barth RJ (1996) In-situ cytokine production by breast-cancer tumour-infiltrating lymphocytes. *Ann Surg Oncol* **3**: 176–184

Campana D, Coustan-Smith E and Janossy G (1988) Double and triple staining methods for studying the proliferative activity of human B and T lymphoid cells. *J Immunol Method* **107**: 79–88

Cavallo F, Martinfontecha A, Bellone M, Heltai S, Gatti E, Tornaghi P, Freschi M, Forni G, Dellabona P and Casorati G (1995) Coexpression of B7-1 and ICAM-1 on tumors is required for rejection and the establishment of a memory response. *Eur J Immunol* **25**: 1154–1162

Chambers CA and Allison JP (1997) Co-stimulation in T cell responses. *Curr Opin Immunol* **9**: 396–404

Coventry BJ, Weeks SC, Heckford SE, Sykes PJ, Bradley J and Skinner JM (1996) Lack of il-2 cytokine expression despite il-2 messenger-rna transcription in tumor-infiltrating lymphocytes in primary human breast-carcinoma – selective expression of early activation markers. *J Immunol* **156**: 3486–3492

Finke JH, Zea AH, Stanley J, Longo DL, Mizoguchi H, Tubbs RR, Wiltrout RH, Oshea JJ, Kudoh S, Klein E, Bukowaski RM and Ochoa AC (1993) Loss of T-cell receptor zeta-chain and p56(lck) in T-cells infiltrating human renal-cell carcinoma. *Cancer Res* **53**: 5613–5616

Hughes KS (1988) Resection of the liver for colorectal-carcinoma metastases – a multi-institutional study of indications for resection. *Surgery* **103**: 278–288

Kawakami Y, Eliyahu S, Delgado CH, Robbins PF, Sakaguchi K, Appella E, Yannelli JR, Adema GJ, Miki T and Rosenberg SA (1994) Identification of a human-melanoma antigen recognized by tumor-infiltrating lymphocytes associated with in-vivo tumor rejection. *Proc Natl Acad Sci USA* **91**: 6458–6462

Kronke M, Leonard WJ, Depper JM and Greene WC (1985) Sequential expression of genes involved in human T lymphocyte growth and differentiation. *J Exp Med* **161**: 1593–1598

Kudoh S, Stanley J, Edinger MG, Tubbs RR, Klein E, Bukowski RM and Finke JH (1994) T-lymphocytes infiltrating renal-cell carcinoma have a reduced expression of transferrin receptor. *Int J Cancer* **58**: 369–375

Lanier LL and Phillips JH (1996) Inhibitory MHC class I receptors on NK cells and T-cells. *Immunol Today* **17**: 86–91

© Cancer Research Campaign 1998

Maeurer MJ, Martin DM, Castelli C, Elder E, Leder G, Storkus WJ and Lotze MT (1995) Host immune-response in renal-cell cancer – interleukin-4 (IL-4) and IL-10 messenger-RNA are frequently detected in freshly collected tumor-infiltrating lymphocytes. *Cancer Immunol Immunother* **41**: 111–121

Miescher S, Whiteside TL, Carrel S and Vonfliedner V (1986) Functional properties of tumor-infiltrating and blood-lymphocytes in patients with solid tumors – effects of tumor-cells and their supernatants on proliferative responses of lymphocytes. *J Immunol* **136**: 1899–1907

Miescher S, Stoeck M, Qiao L, Barras C, Barrelet L and Vonfliedner V (1988) Proliferative and cytolytic potentials of purified human tumor-infiltrating lymphocytes – impaired response to mitogen-driven stimulation despite T-cell receptor expression. *Int J Cancer* **42**: 659–666

Mizoguchi H, Oshea JJ, Longo DL, Loeffler CM, McVicar DW and Ochoa AC (1992) Alterations in signal transduction molecules in lymphocytes from tumor-bearing mice. *Science* **258**: 1795–1798

Nakagomi H, Petersson M, Magnusson I, Juhlin C, Matsuda M, Mellstedt H, Taupin JL, Vivier E, Anderson P and Kiessling R (1993) Decreased expression of the signal-transducing zeta-chains in tumor-infiltrating T-cells and NK cells of patients with colorectal-carcinoma. *Cancer Res* **53**: 5610–5612

Nakagomi H, Pisa P, Pisa EK, Yamamoto Y, Halapi E, Backlin K, Juhlin C, and Kiessling R (1995) Lack of interleukin-2 (IL-2) expression and selective expression of IL-10 messenger RNA in human renal-cell carcinoma. *Int J Cancer* **63**: 366–371

O'Connell J, O'Sullivan GC, Collins JK and Shanahan F (1996) The fas counterattack – fas-mediated T-cell killing by colon-cancer cells expressing fas ligand. *J Exp Med* **184**: 1075–1082

Rivoltini L, Kawakami Y, Sakaguchi K, Southwood S, Sette A, Robbins PF, Marincola FM, Salgaller ML, Yannelli JR, Appella E and Rosenberg SA (1995) Induction of tumor-reactive CTL from peripheral-blood and tumor-infiltrating lymphocytes of melanoma patients by in-vitro stimulation with an immunodominant peptide of the human-melanoma antigen mart-1. *J Immunol* **154**: 2257–2265

Robbins PF and Kawakami Y (1996) Human tumor antigens recognised by T cells. *Curr Opin Immunol* **8**: 628–636

Robey E and Allison JP (1995) T-cell activation – integration of signals from the antigen receptor and costimulatory molecules. *Immunol Today* **16**: 306–310

Rosenberg SA, Yannelli JR, Yang JC, Topalian SL, Schwartzentruber DJ, Weber JS, Parkinson DR, Seipp CA, Einhorn JN and White DE (1994) Treatment of patients with metastatic melanoma with autologous tumor-infiltrating lymphocytes and interleukin-2. *J Natl Cancer Inst* **86**: 1159–1166

Sanderson CJ (1976) The uptake and retention of chromium by cells. *Transplantation* **21**: 526–529

Sayegh MH, Akalin E, Hancock WW, Russell ME, Carpenter CB, Linsley PS and Turka LA (1995) CD28-B7 blockade after alloantigenic challenge in vivo inhibits Th1 but spares Th2 cytokines. *J Exp Med* **181**: 1869–1874

Schendel DJ, Gansbacher B, Oberneder R, Kriegmair M, Hofstetter A, Riethmuller G and Segurado OG (1993) Tumor-specific lysis of human renal-cell carcinomas by tumor-infiltrating lymphocytes. 1. HLA A2-restricted recognition of autologous and allogeneic-tumor lines. *J Immunol* **151**: 4209–4220

Schwartz RH (1992) Costimulation of T lymphocytes: the role of CD28, CTLA-4, and B7/BB1 in interleukin-2 production and immunotherapy. *Cell* **71:** 1065–1068

Shimizu Y, Iwatsuki S, Herberman RB and Whiteside TL (1990) Clonal analysis of tumour infiltrating lymphocytes from human primary and metastatic liver tumours. *Int J Cancer* **46**: 878–883

Townsend SE and Allison JP (1993) Tumor rejection after direct costimulation of CD8 + T-cells by B7-transfected melanoma-cells. *Science* **259**: 368–370

Townsend SE, Su FW, Atherton JM and Allison JP (1994) Specificity and longevity of antitumor immune-responses induced by B7-transfected tumors. *Cancer Res* **54**: 6477–6483

van der Bruggen P, Traversari C, Chomez P, Lurquin C, De Plaen E, van den Eynde B, Knuth A and Boon T (1991) A gene encoding an antigen recognised by cytolytic T lymphocytes on a human melanoma. *Science* **2**: 1643–1647

Yannelli JR (1991) The preparation of effector cells for use in the adoptive cellular therapy of cancer. *J Immunol Method* **139**: 1–16

Yoong KF and Adams DH (1996) Tumour infiltrating lymphocytes: insights into tumour immunology and potential for immunotherapy. *J Clin Mol Pathol* **49**: M267–M277

© Cancer Research Campaign 1998